# Angiogenesis Inhibitors and Arterial Dissection or Aneurysm in Patients With Metastatic Colorectal Cancer

**DOI:** 10.1001/jamanetworkopen.2025.46960

**Published:** 2025-12-04

**Authors:** Allison Singier, Ana Jarne Munoz, Sandy Maumus-Robert, Xavier Bérard, Antoine Pariente, Julien Bezin, Emmanuelle Kempf, Pernelle Noize

**Affiliations:** 1 Univ. Bordeaux, INSERM, BPH, U1219, AHeaD Team, Bordeaux, France; 2 CHU de Bordeaux, Pôle de chirurgie, Service de chirurgie vasculaire et endovasculaire, Bordeaux, France; 3 CHU de Bordeaux, Pôle de santé publique, Service de pharmacologie médicale, Bordeaux, France; 4 AP-HP, Hôpital Henri-Mondor, Service d’oncologie médicale, Créteil, France

## Abstract

**Importance:**

Although pharmacovigilance signals and a strong pathophysiological rationale have suggested a potential risk of arterial dissections or aneurysms associated with angiogenesis inhibitors, this association deserves to be further investigated through clinical practice evidence studies.

**Objective:**

To evaluate the association between exposure to angiogenesis inhibitors and the occurrence of arterial dissections or aneurysms in patients treated for metastatic colorectal cancer (mCRC).

**Design, Setting, and Participants:**

A nested case-control study was conducted within a cohort of adults initiating targeted therapy (angiogenesis or epidermal growth factor receptor inhibitors) for mCRC between January 1, 2012, and December 31, 2017. Data were analyzed from April 2021 through August 2023. Data were drawn from the French nationwide Système National des Données de Santé database, which combines health insurance and hospital discharge records. Cases of arterial dissection or aneurysm were identified through 2019 and matched with up to 10 controls by age, sex, and time since cohort entry.

**Exposures:**

The angiogenesis inhibitors indicated for mCRC in France (ie, bevacizumab, aflibercept, ramucirumab, and regorafenib) were considered. Exposure was defined using 3 criteria: exposure at any point (exposed vs unexposed), recency (current, past, or unexposed), and cumulative duration of exposure (quartiles). Exposure periods were estimated using recommended administration schedules or drug elimination times.

**Main Outcomes and Measures:**

The primary outcome was incident hospitalization for arterial dissection or aneurysm, identified through hospital discharge diagnoses. Conditional logistic regression models were applied to estimate the association between exposure to angiogenesis inhibitors and the occurrence of arterial dissections or aneurysms, expressed as odds ratios (ORs) with 95% CIs.

**Results:**

Of the 34 733 patients included in the cohort, 195 incident cases (0.6%) were matched with 1950 controls. The study population (2145 patients) included 1562 male patients (72.8%); the median (IQR) age was 69 (63-73) years for cases and 68 (62-73) years for controls. Considering exposure at any point, 141 cases (72.3%) and 1381 controls (70.8%) were exposed to angiogenesis inhibitors; after adjustment for cardiovascular risk level, no association was observed between exposure and the occurrence of arterial events (OR, 1.07; 95% CI, 0.75-1.52). No associations were found with either recency or cumulative duration of exposure, regardless of the exposure estimation method.

**Conclusions and Relevance:**

In this case-control study of arterial dissections or aneurysms in patients receiving targeted therapy for mCRC, the lack of association with angiogenesis inhibitor exposure was reassuring, given the established benefits of these drugs, particularly bevacizumab, for this indication.

## Key Points

**Question:**

Are angiogenesis inhibitors associated with arterial dissections or aneurysms among patients with metastatic colorectal cancer?

**Findings:**

This nested case-control study of 2145 patients from a cohort of patients initiating targeted therapy (angiogenesis or epidermal growth factor receptor inhibitors) for metastatic colorectal cancer, found a low incidence of arterial dissections or aneurysms (0.6%) and no association between exposure to angiogenesis inhibitors and the occurrence of these events after adjustment for cardiovascular risk level.

**Meaning:**

These findings suggest that the established benefits of antiangiogenic drugs in metastatic colorectal cancer appear to outweigh the potential risk of arterial dissections and aneurysms in these patients.

## Teaser

This case-control study evaluates the association between exposure to angiogenesis inhibitors and the occurrence of arterial dissections or aneurysms in patients treated for metastatic colorectal cancer.

## Introduction

Angiogenesis, the formation of new blood vessels from preexisting vasculature, is a crucial process in cancer progression.^1^ Investigating the signaling pathways involved in angiogenesis, particularly those centered on vascular endothelial growth factor (VEGF), has driven the development of angiogenesis inhibitors for cancer therapy.^2,3^ These inhibitors impede VEGF activation by either blocking its binding to cell-surface receptors or disrupting intracellular pathways. Currently, angiogenesis inhibitors are used across various oncology subfields, including gastroenterology, urology, and nephrology.^4^

Although their mechanism of action suggests a potential for arterial wall damage,^5^ data assessing the link between angiogenesis inhibitors and arterial dissections or aneurysms are limited to case reports, case series,^6^ and pharmacovigilance studies.^7,8,9,10^ In December 2018, Health Canada issued a caution about this risk with VEGF receptor tyrosine kinase inhibitors,^11^ followed by similar alerts from regulatory authorities in the European Union, US, and United Kingdom between July 2019 and July 2020. The European Medicines Agency’s Pharmacovigilance Risk Assessment Committee also recommended updating product labels for all angiogenesis inhibitors.^12,13,14^ Given these signals and the limitations of pharmacovigilance studies that rely on spontaneous adverse event reporting, a pharmacoepidemiological study was warranted to further explore this association.

In a 2021 pharmacovigilance study, Guyon et al^8^ reported that implicated drugs were predominantly used in gastrointestinal cancers. Specifically, bevacizumab—a widely used first-line treatment in metastatic colorectal cancer (mCRC)—was identified in 45% of reported cases of arterial dissections or aneurysms. Given this, and considering that other targeted therapies are available for patients with mCRC at similar disease stages as those treated with angiogenesis inhibitors,^15^ the present study aimed to examine the association between exposure to angiogenesis inhibitors and the occurrence of arterial dissections or aneurysms in patients receiving treatment for mCRC.

## Methods

This study was conducted and reported according to Strengthening the Reporting of Observational Studies in Epidemiology (STROBE) reporting guidelines.^16^ In compliance with French regulations, neither ethics committee approval nor informed consent was required, as this observational study utilized anonymized data from French medico-administrative databases.

### Data Source

This study used the Système National des Données de Santé (SNDS), a comprehensive database integrating national health insurance records with hospital discharge data, capturing information on more than 99% of the French population. The SNDS provides anonymous data on all outpatient health care reimbursements, including pharmaceuticals, consultations, and laboratory tests. However, detailed data on prescriptions, results from medical procedures, and laboratory tests are not available. The SNDS includes information on sociodemographic characteristics, hospital diagnoses, in-hospital administration of high-cost drugs (including most targeted therapies used in oncology), and records of severe long-term diseases eligible for full health care reimbursement. Diagnoses are coded according to the *International Statistical Classification of Diseases and Related Health Problems, Tenth Revision (ICD-10)*, and drugs are coded according to the Anatomical Therapeutic Chemical (ATC) classification. Further details on the SNDS have been described previously.^17^ This analysis focused specifically on individuals covered by the main health insurance scheme, which includes approximately 87% of the French population, such as salaried workers, retired salaried workers, and their dependents.

### Study Design and Setting

We conducted a nested case-control study within a cohort of adults who initiated targeted therapy for mCRC—angiogenesis inhibitors or epidermal growth factor receptor (EGFR) inhibitors—between January 1, 2012, and December 31, 2017, with no history of these therapies dispensing. The angiogenesis inhibitors studied included bevacizumab (ATC code L01XC07), aflibercept (ATC code L01XX44), ramucirumab (ATC code L01XC21), and regorafenib (ATC code L01XE21), whereas the EGFR inhibitors included cetuximab (ATC code L01XC06) and panitumumab (ATC code L01XC08). The date of the first targeted therapy dispensing was set as the cohort entry date, and each patient was followed-up until the earliest of a study outcome, death, loss of available information, or December 31, 2019.

Eligibility criteria for the source cohort included enrollment in the main health insurance scheme and no history of arterial dissection or aneurysm in the 2 years before cohort entry date. To confirm the use of targeted therapies for mCRC, at least 1 *ICD-10* code for colorectal cancer (C18-C20) had to be recorded, as either hospital discharge diagnosis (primary, related, or associated) or a registered severe long-term condition, from 2010 to the end of follow-up.

### Selection of Cases and Controls

Cases were identified from the source cohort as patients with a first hospitalization for arterial dissection or aneurysm, defined as having either primary, related, or associated hospital discharge diagnoses using *ICD-10* codes I71, I72.2, I72.3, I72.8, I60 (excluding I60.8), I67.1, I72.0, I72.5, and I72.6 based on a previously published algorithm.^18^ The date of this initial hospital admission for arterial dissection or aneurysm was defined as the index date.

All patients in the source cohort were eligible to serve as potential controls. Each case was matched at the index date with up to 10 controls selected through risk set sampling; matching factors were sex, age at the index date (18-50, 51-65, 66-75, 76-85, or ≥86 years), and follow-up duration within the source cohort (0-6, 7-12, 13-24, 25-36, or ≥37 months).

### Exposure to Angiogenesis Inhibitors

**Figure 1.**
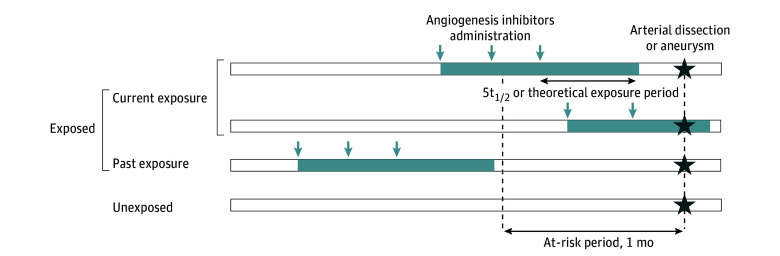
Definition of Angiogenesis Inhibitors Exposure Periods Figure shows how exposure to angiogenesis inhibitors was defined. Patients were considered exposed if they received at least 1 dispensing between cohort entry and the index date (main analysis). Among exposed patients, current exposure refers to at least 1 day of exposure during the month before or on the index date; past exposure refers to exposure that ended more than 1 month before the index date. Exposure periods associated with each dispensing were estimated using 2 approaches: (1) a theoretical exposure period based on the recommended administration schedule for each drug, and (2) an elimination period corresponding to 5 elimination half-lives (5t_1/2_). Patients with no dispensing during the study period were considered unexposed (reference group).

All angiogenesis inhibitors indicated for mCRC in France were considered: bevacizumab, aflibercept, ramucirumab, and regorafenib. Exposure periods were estimated using 2 approaches (eTable 1 in Supplement 1). The first used the recommended administration schedule for each drug. To account for possible delays in treatment, this theoretical period was doubled (eg, for bevacizumab recommended every 2 weeks, the theoretical exposure period was set at 4 weeks after each dispensing). The second approach estimated exposure by applying an elimination time equal to 5 elimination half-lives, during which approximately 97% of the drug is expected to be eliminated. Three definitions of exposure to angiogenesis inhibitors were used for the analyses (Figure 1): (1) exposure at any point (main analysis), defined as at least 1 dispensing between cohort entry and the index date; (2) recency of exposure, classified as either current exposure (at least 1 day of exposure during the month preceding the index date or exposure ongoing at the index date) or past exposure (all days of exposure before the month preceding the index date); and (3) cumulative duration of exposure, expressed as the total number of days exposed since cohort entry, divided into quartiles. Patients with no dispensing of angiogenesis inhibitors during this period were classified as never exposed and served as the reference group.

### Statistical Analysis

Data were analyzed from April 2021 through August 2023 using SAS Enterprise Guide software version 9.4 (SAS Institute). Conditional logistic regression models were applied to estimate the association between exposure to angiogenesis inhibitors and the occurrence of arterial dissections or aneurysms, expressed as odds ratios (ORs) with 95% CIs. Statistical significance was defined as a 95% CI that did not include the null value of 1. All models were adjusted for cardiovascular risk level, on the basis of the presence of cardiovascular diseases or risk factors in the 2 years preceding the index date. Cardiovascular risk was categorized into 4 levels according to the European Society of Cardiology classification^19^ (eTable 2 in Supplement 1): level 1, very high cardiovascular risk (established atherosclerotic cardiovascular disease, including ischemic heart disease, transient ischemic attack, ischemic stroke, coronary revascularization, peripheral arteriopathy, or use of nitrate derivatives or other anti-ischemic drugs as proxies for stable angina); level 2, high cardiovascular risk (no atherosclerotic cardiovascular disease, but presence of chronic kidney disease, complicated diabetes, or uncomplicated diabetes with at least 1 additional cardiovascular risk factor, such as hypertension, obesity, tobacco or alcohol-related diseases); level 3, moderate cardiovascular risk (hypertension, obesity, tobacco or alcohol-related diseases, treatment with antiplatelet or anticoagulant drugs, or uncomplicated diabetes without additional risk factors); and level 4, low cardiovascular risk (absence of any of the above conditions). A sensitivity analysis was conducted by restricting cases to those with arterial dissection or aneurysm identified as the primary hospital discharge diagnosis.

## Results

### Characteristics of the Study Population

**Figure 2.**
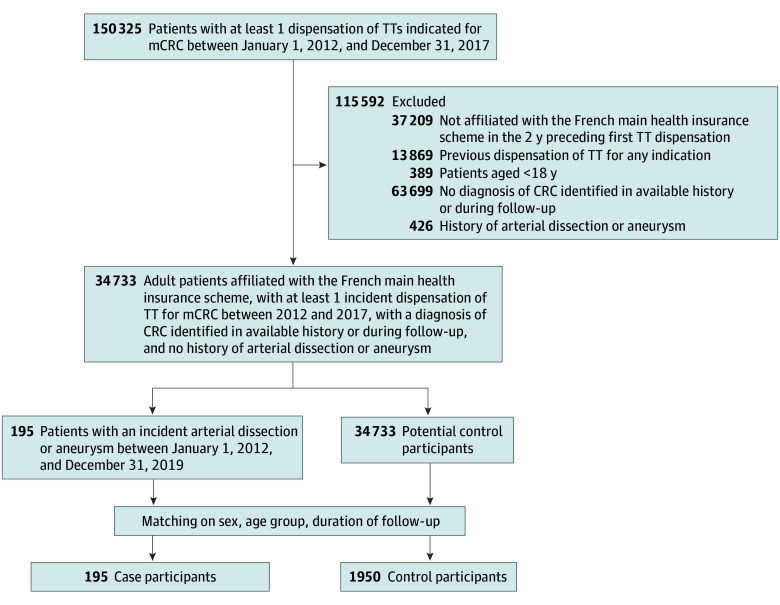
Flowchart of Cases and Controls Selection CRC indicates colorectal cancer; mCRC, metastatic colorectal cancer; TT, targeted therapy.

Of 150 325 patients who received at least 1 dispensing of an angiogenesis or EGFR inhibitor of interest between January 1, 2012, and December 31, 2017, 34 733 were included in the source cohort. Among these, 195 (0.6%) were identified as incident cases of arterial dissection or aneurysm, involving the aorta or iliac arteries (118 cases [60.5%]), visceral arteries (42 cases [21.5%]), intracranial arteries (39 cases [20.0%]), and cervical arteries (5 cases [2.6%]). These cases were matched to 1950 controls (Figure 2).

The study population (2145 patients) included 1562 male patients (72.8%) and 583 female patients (27.2%). The median (IQR) age was 69 (63-73) years for cases and 68 (62-73) years for controls (Table). Clinical characteristics assessed in the 2 years preceding cohort entry showed that 69 cases (35.4%) had a high to very high cardiovascular risk, compared with 582 controls (29.9%). Very high cardiovascular risk was more frequently observed among cases (42 patients [21.5%]) than controls (265 patients [13.6%]). Cases were also more likely than controls to have peripheral arterial disease (30 patients [15.4%] vs 81 patients [4.2%]), tobacco-related diseases (38 patients [19.5%] vs 215 patients [11.0%]), and treatment with antiplatelet drugs (59 patients [30.3%] vs 436 patients [22.4%]) or lipid-lowering drugs (85 patients [43.6%] vs 746 patients [38.3%]).

**Table.** Characteristics of the Study Population at Source Cohort Entry Date

Characteristics	Patients, No. (%) (N = 2145)	
	Cases (n = 195)	Controls (n = 1950)
Demographic characteristics		
Sex		
Female	53 (27.2)	530 (27.2)
Male	142 (72.8)	1420 (72.8)
Age, median (IQR), y	69 (63-73)	68 (62-73)
Cardiovascular characteristics^a^		
Cardiovascular risk level		
Very high risk	42 (21.5)	265 (13.6)
High risk	27 (13.8)	317 (16.3)
Moderate risk	105 (53.8)	1150 (59.0)
Low risk	21 (10.8)	218 (11.2)
Ischemic heart disease	15 (7.7)	163 (8.4)
Peripheral artery disease	30 (15.4)	81 (4.2)
Ischemic stroke	0	11 (0.6)
Transient ischemic attack	0	3 (0.2)
Hypertension or use of antihypertensive drugs	119 (61.0)	1190 (61.0)
Cardiac failure	7 (3.6)	60 (3.1)
Dysrhythmias	24 (12.3)	244 (12.5)
Use of lipid-lowering drugs	85 (43.6)	746 (38.3)
Use of anticoagulant drugs	116 (59.5)	1225 (62.8)
Use of antiplatelet drugs	59 (30.3)	436 (22.4)
Use of nitrate derivatives or other anti-ischemic drugs	7 (3.6)	84 (4.3)
Other clinical characteristics^a^		
Chronic kidney disease	8 (4.1)	26 (1.3)
Diabetes	28 (14.4)	379 (19.4)
Obesity	17 (8.7)	194 (9.9)
Tobacco-related diseases	38 (19.5)	215 (11.0)
Alcohol-related diseases	15 (7.7)	84 (4.3)
Obstructive sleep apnea	6 (3.1)	58 (3.0)
Chronic obstructive pulmonary disease	23 (11.8)	135 (6.9)
Use of antibiotics	147 (75.4)	1506 (77.2)
Quinolone	61 (31.3)	574 (29.4)
Use of systemic corticoids	94 (48.2)	897 (46.0)
Cancer treatment history^a^		
Other anticancer drugs (including chemotherapy)	123 (63.1)	1320 (67.7)
Radiotherapy	11 (5.6)	125 (6.4)
Primary tumor surgery	107 (54.9)	1168 (59.9)
Metastases surgery	31 (15.9)	385 (19.7)
Focal treatments for metastatic lesions^b^	10 (5.1)	77 (4.0)

^a^ Evaluated within 2 years prior to cohort entry.

^b^ Refers to chemoembolization, radioembolization, intra-arterial chemotherapy, and radiofrequency.

Regarding prior cancer treatments, cases were slightly less likely than controls to have undergone chemotherapy or surgery for the primary tumor or metastases. However, cases and controls showed similar frequencies for radiotherapy (11 patients [5.6%] vs 125 patients [6.4%]) and focal treatments for metastatic lesions (10 patients [5.1%] vs 77 patients [4.0%]).

### Association Between Angiogenesis Inhibitors and Arterial Dissections or Aneurysms

**Figure 3.**
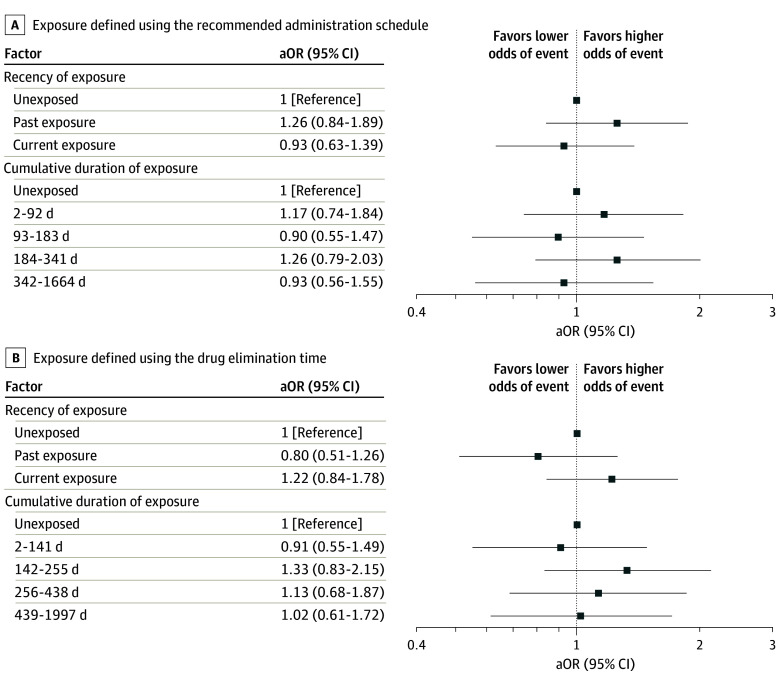
Association Between Exposure to Angiogenesis Inhibitors and Arterial Dissections or Aneurysms in Patients With Metastatic Colorectal Cancer Forest plots present the results of secondary analyses assessing the association between exposure to angiogenesis inhibitors and the risk of arterial dissections or aneurysms in patients with metastatic colorectal cancer. A, Exposure defined using the recommended administration schedule. B, Exposure defined using the drug elimination time. aOR indicates adjusted odds ratio.

When considering exposure at any point (eg, at least 1 dispensing between cohort entry and the index date), 141 cases (72.3%) and 1381 controls (70.8%) were classified as exposed to angiogenesis inhibitors. No association was observed with the occurrence of arterial dissections or aneurysms in this main analysis (crude OR, 1.09; 95% CI, 0.76-1.54; OR adjusted on the cardiovascular risk level, 1.07; 95% CI, 0.75-1.52). Similarly, secondary analyses examining recency and cumulative duration of exposure did not reveal any associations with arterial dissections or aneurysms (Figure 3; eTable 3 in Supplement 1). The absence of association was consistent across the different methods used to estimate exposure periods. Findings from the sensitivity analysis, which was restricted to cases of arterial dissection or aneurysm identified as the primary hospital discharge diagnosis, were consistent with the main analysis (crude OR, 0.74; 95% CI, 0.43-1.28; OR adjusted on the cardiovascular risk level, 0.70; 95% CI, 0.40-1.23).

## Discussion

The association between exposure to angiogenesis inhibitors and the occurrence of arterial dissections or aneurysms was evaluated in the specific context of mCRC through a nested case-control study using the French nationwide SNDS database. No association was found after adjustment for cardiovascular risk level, with a low incidence of events in the source cohort (195 cases among 34 733 patients over the 2012-2019 period; ie, 0.6%).

This study provides reassuring evidence regarding the association between angiogenesis inhibitors exposure and arterial dissections or aneurysms in patients treated for mCRC. To our knowledge, no comparable study has been published. Recently, a South Korean cohort study^20^ and a Taiwanese nested case-control study,^21^ both based on medico-administrative databases, have been reported. Nevertheless, they specifically investigated this association for oral angiogenesis-targeting protein kinase inhibitors, which are more easily identifiable in medico-administrative databases, and in populations of patients with various cancer types. Both studies reported an association, with a relative risk of 1.48 (95% CI, 1.08-2.02) compared with capecitabine in the study by Kang et al^20^ and an OR of 2.00 (95% CI, 1.41-2.84) compared with nonexposure in the study by Wu et al.^21^ Beyond the restriction to the class of oral protein kinase inhibitors, interpreting these results is challenging because of the heterogeneity of the patient populations studied. As highlighted in the introduction, angiogenesis inhibitors are indicated for a wide range of cancers, leading to substantial variability in the alternative therapeutic options available. Furthermore, in some cancers, such as renal cancer, there are few, if any, alternatives to angiogenesis inhibitors, making it difficult to establish a reference group for estimating the association between exposure to these drugs and the outcomes of interest. For example, in the study by Kang et al,^20^ the only active comparator used was capecitabine, whose approved indications (colorectal, gastric, and breast cancers) do not fully overlap with those of the 7 angiogenesis inhibitors investigated (sorafenib, regorafenib, vandetanib, sunitinib, lenvatinib, axitinib, and pazopanib). In addition to differences in therapeutic management, patients may have varying baseline risks of arterial dissections or aneurysms depending on cancer type. Distinct pathophysiological mechanisms involved in cancer development and progression, such as inflammation or oxidative stress, may differentially influence vascular risks. Although both studies used optimal adjustments for medico-administrative database analyses, their findings do not clarify whether the risk differs, or is absent, for specific cancers or subgroups, such as mCRC.

### Strengths and Limitations

This study uses the SNDS database, which provides nationally representative clinical practice data and comprehensive information on drug exposure, hospitalization diagnoses, and surgical procedures. A key methodological strength of this study is the use of a homogeneous population of patients treated for a single indication, mCRC, by restricting the nested case-control analysis to those initiating targeted therapy, either angiogenesis or EGFR inhibitors. In mCRC, the choice of therapeutic strategy—targeted therapy combined with conventional chemotherapy, conventional chemotherapy alone, or supportive care—is heavily influenced by tumor-related and patient-related factors, including mutational tumor status, patient comorbidities and functional status. Thus, identifying cases and selecting controls within a population with a similar a priori likelihood of angiogenesis inhibitors exposure minimizes confounding due to disparities in cardiovascular comorbidities or cancer characteristics, such as inflammation and the tumor microenvironment. However, patients with altered cardiovascular profiles and *RAS*-altered mCRC may have been underrepresented in the source cohort, as they are ineligible for anti-EGFR therapy and less likely to receive angiogenesis inhibitors as first-line treatment. Despite this, the sociodemographic and clinical characteristics of the source cohort were consistent with expected profiles, providing reassurance regarding its overall quality.

Although focusing on patients initiating targeted therapy for mCRC is a major strength, it also required some methodological compromises. The relatively small size of the source cohort did not allow the use of an existing algorithm that combines hospital diagnoses, procedures, and vital status, as it identified too few events. As a result, event identification relied solely on hospitalization diagnosis codes. This approach led to a sensitive event definition that may have included false-positives for arterial dissections and aneurysms. However, any misclassification is unlikely to have differed between patients treated with or without angiogenesis inhibitors. Supporting this, the sensitivity analysis, which was limited to cases with a primary discharge diagnosis, produced results consistent with the main analysis.

Using primary, related, and associated discharge diagnoses, an incidence of 0.6% was observed in the source cohort during the 2012 to 2019 period. Although comparing this incidence with other epidemiological data is challenging, it aligns with findings from the 2 recently published studies on the topic.^20,21^ In the cohort from Kang et al,^20^ 109 arterial dissections or aneurysms were identified among 27 535 patients initiating 7 angiogenesis-targeting kinase inhibitors for various cancer indications during 2010 to 2021, corresponding to an incidence of 0.4%.^20^ Similarly, in the nested case-control study from Wu et al,^21^ 1461 cases of aortic dissections or aneurysms were identified among a cohort of 417 302 patients with cancers for which 3 angiogenesis-targeting kinase inhibitors are indicated (2012-2019), corresponding to an incidence of 0.35%.^21^ In addition, the limited number of cases identified in the present study precluded a detailed characterization of early-onset events. Identifying and profiling patients at higher risk of arterial dissections or aneurysms within the first months of mCRC-targeted therapy initiation could be clinically useful for considering alternative therapeutic options or reinforcing early clinical monitoring.

Exposure was primarily defined as receiving at least 1 dispensing of angiogenesis inhibitors since cohort entry, offering a broad view. While this approach does not account for time-dependent exposure dynamics, additional analyses investigating the recency and cumulative duration of exposure produced consistent results, supporting the absence of association. Although these exposure subcategories could raise concerns about statistical power, all of them included sufficient number of participants, and 95% CIs remained relatively centered around the estimated OR. Importantly, 2 complementary methods were used to define exposure periods, enhancing the robustness of exposure assessment: one based on the recommended administration schedule, which tends to minimize exposure duration, and the other based on elimination half-life, which tends to maximize it.

In addition, confounding bias was minimized using case-control matching, combined with model adjustment accounting for age, sex, and cardiovascular risk level, factors associated with both the likelihood of receiving angiogenesis inhibitors and the risk of arterial dissections or aneurysms. This approach also helped balance several relevant characteristics and risk factors between cases and controls, including clinical characteristics, drug treatments, or therapeutic or surgical procedures. Although peripheral arterial disease and tobacco-related diseases were not perfectly balanced, they were accounted for through adjustment, as they were components of the cardiovascular risk score. Despite these efforts, residual confounding cannot be entirely excluded, particularly for time-varying factors such as hypertension, which could not be fully captured using SNDS data. Moreover, matching on a specific arterial dissection or aneurysm disease risk score derived from a previous study^18^ could have further improved the baseline risk comparability between cases and controls. However, this strategy was limited by the small number of events and the low proportion of unexposed patients in the source cohort. The 2012 to 2019 time frame provides a pre–COVID-19 context, avoiding care disruptions that affected oncology. The inclusion period (2012 to 2017) also predates regulatory warnings on angiogenesis inhibitors, reducing notoriety or screening bias and offering a clearer baseline risk estimate. Importantly, the data remain relevant, as angiogenesis inhibitors—especially bevacizumab—continue to be standard in first-line and subsequent mCRC treatments.

## Conclusions

In this case-control study of the association between exposure to angiogenesis inhibitors and the occurrence of arterial dissections and aneurysms in the specific clinical context of mCRC, reassuring evidence was found regarding the low incidence of these events and the absence of a significant association in this indication. These findings are relevant to routine clinical practice, given the well-established survival benefits of angiogenesis inhibitors in mCRC, especially bevacizumab, including in older patients with cardiovascular comorbidities,^22^ as well as their increasing use beyond first-line treatment. However, these reassuring findings should not diminish the importance of individual patient monitoring, particularly for those with a high-risk profile for arterial events, such as smokers with peripheral arterial disease.

## Notes

Supplement 1. eTable 1. Key characteristics to build exposure periods to angiogenesis inhibitors eTable 2. Codes for identification of cardiovascular risk level eTable 3. Association between exposure to angiogenesis inhibitors and arterial dissections or aneurysms in mCRC patients, by exposure period estimation method

Supplement 2. Data Sharing Statement
