# Supplementary material for: Angiogenesis Inhibitors and Arterial Dissection or Aneurysm in Patients With Metastatic Colorectal Cancer
Source: JAMA Netw Open. 2025 Dec 4;8(12):e2546960. doi: 10.1001/jamanetworkopen.2025.46960 (PMC12679322; doi:10.1001/jamanetworkopen.2025.46960)
Supplement: Supplement 1. — eTable 1. Key characteristics to build exposure periods to angiogenesis inhibitors eTable 2. Codes for identification of cardiovascular risk level eTable 3. Association between exposure to angiogenesis inhibitors and arterial dissections or aneurysms in mCRC patients, by exposure period estimation method [file jamanetwopen-e2546960-s001.pdf]

Supplemental Online Content

Singier A, Jarne Munoz A, Maumus-Robert S, et al. Angiogenesis inhibitors and arterial dissection or aneurysm in patients with metastatic colorectal cancer. *JAMA Netw Open*. 2025;8(12):e2546960. doi:10.1001/jamanetworkopen.2025.46960

eTable 1. Key characteristics to build exposure periods to angiogenesis inhibitors

eTable 2. Codes for identification of cardiovascular risk level

eTable 3. Association between exposure to angiogenesis inhibitors and arterial dissections or aneurysms in mCRC patients, by exposure period estimation method

This supplemental material has been provided by the authors to give readers additional information about their work.

© 2025 Singier A et al. *JAMA Network Open*.

eTable 1. Key characteristics to build exposure periods to angiogenesis inhibitors

Angiogenesis inhibitor	Half-life ($t_{1/2}$)	Elimination time ($5t_{1/2}$)	Recommended administration schedule	Theoretical exposure period
Bevacizumab	20 days	100 days	Every 2 weeks	28 days
Aflibercept	6 days	30 days	Every 2 weeks	28 days
Ramucirumab	14 days	70 days	Every 2 weeks	28 days
Regorafenib	2.5 days	12.5 days	Continuous for 3 weeks followed by a 1-week break	56 days

© 2025 Singier A et al. *JAMA Network Open*.

eTable 2. Codes for identification of cardiovascular risk level

	Source	Codes
**Very high cardiovascular risk – *established atherosclerotic cardiovascular disease (ASCVD)***		
Ischemic heart disease	Hospital discharge diagnosis (ICD-10) or registration for long-term disease (ICD-10)	I20-I25
Transient ischemic attack	Hospital discharge diagnosis (ICD-10) or registration for long-term disease (ICD-10)	G450, G451, G452, G453, G458, G459
Ischemic stroke	Hospital discharge diagnosis (ICD-10) or registration for long-term disease (ICD-10)	I63
Coronary revascularization	Medical procedure (CCAM)	DDAA002, DDAF001, DDAF003, DDAF004, DDAF006-DDAF010, DDFF001, DDFF002, DDPF002, DDMA002-DDMA009, DDMA011-DDMA013, DDMA015-DDMA038
Peripheral artery disease	Hospital discharge diagnosis (ICD-10) or registration for long-term disease (ICD-10)	I70-I74
Nitrates or anti-ischemic drugs	Drug dispensing (ATC)	C01DA, C01DX12, C01DX16
**High cardiovascular risk – *no ASCVD but presence of chronic renal failure, complicated diabetes, or uncomplicated diabetes with at least one additional cardiovascular risk factor***		
Chronic kidney disease	Hospital discharge diagnosis (ICD-10) or registration for long-term disease (ICD-10)	N04, N07, N08, N11, N14, N15, N16, N18, N19, N25, N26, N27, N28, N29, Q61, Z94.0
	Medical procedure (CCAM)	JVJB001, JVJF004, JVJF008, JVRP004, JVRP007, JVRP008, YYYY007, JAEA003, HNEA002
	Hospital diagnosis related group	11K021, 11K022, 11K023, 11K024, 11K02J, 28Z01Z, 28Z02Z, 28Z03Z, 28Z04Z, 28Z05Z, 28Z06Z, 27C06Z, 27C061, 27C062, 27C063, 27C064, 24M39Z, 11M171, 11M172, 11M173, 11M174
	Drug dispensing (ATC)	L04AA06, L04AA10, L04AA18, L04AD01, L04AD02, L04AX01
	French reimbursement code	2121, 2122, 2123, 2124, 2125, 2126, 2127, 2128, 2129, 2131, 2132, 2134, 2135, 2136, 2137, 2138, 2139, 2140, 2142, 2143, 2144, 2145, 2146, 2147, 2334
Diabetes with complications	Hospital discharge diagnosis (ICD-10)	E102-E108, E112-E118, E122-E128, E132-E138, E142-E148 or E10-E14 and at least one of the following codes: G590, G632, G730, G990, H280, H360, I792, L97, M142, M146, N083
Diabetes without complications	Hospital discharge diagnosis (ICD-10) or registration for long-term disease (ICD-10)	E10-E14
	Drug dispensing (ATC)	A10A, A10B (excluding A10BX06)

© 2025 Singier A et al. *JAMA Network Open*.

eTable 2. Codes for identification of cardiovascular risk level (continued)

	Source	Codes
Hypertension or antihypertensive drugs	Hospital discharge diagnosis (ICD-10) or registration for long-term disease (ICD-10)	I10-I13, I15, I674
	Drug dispensing (ATC)	C02AB, C02AC, C02CA, C02DC, C02LA01, C03, C07, C08, C09, C10BX03
Obesity	Hospital discharge diagnosis (ICD-10)	E65-E66
	Medical procedure (CCAM)	HFCC003, HFCA001, HFMC007, HFMA009, HFFC018, HFFA011, HFFC004, HFFA001, HGCC027, HGCA009, HFMA011, HFMC008
Tobacco-related diseases	Hospital discharge diagnosis (ICD-10) or registration for long-term disease (ICD-10)	F17, J41, J42, J43, J44, F17, T65.2, Z50.8, I73.1, Z58.7, Z71.6, Z72.0, J41, J42, J43, J44, J96.1
	Drug dispensing (ATC)	N07BA, N06AX12, R03BB05, R03BB04, R03BB07, R03BB06, R03AC18, R03AC19, R03AL03, R03AL04, R03AL05, R03AL06, R03AL08, R03AL09
Alcohol-related diseases	Hospital discharge diagnosis (ICD-10) or registration for long-term disease (ICD-10)	F10, K70, T51, E24.4, F10, G31.2, G62.1, G72.1, I42.6, K29.2, K70, K86.0, T51, Z50.2, Z71.4, Z72.1, C70, C71, C79.3, C79.4, D32, D33, D42, D43, G04, G05, G06, G09, G12, G13, G24, G25, G26, G31, G32, G35, G36, G37, G46, G80, G81, G82, G83, G91, G93, G95
	Drug dispensing (ATC)	N07BB, M03BX01
	French biology code	0519
	French medical device code	4101353, 4164566, 4184899, 4169670, 4107723, 4118193, 4122473, 4179540, 4111854, 4147668, 4130136, 4122757, 4174323, 4159619, 4195615, 4142530, 4183434, 4168966, 4113920, 4252810, 4255116, 4263950, 4261051, 4233570, 4325302, 4371408, 4342654, 4327382, 4329040, 4324739, 4300348, 4321630, 4375613, 4375116, 4371555, 4359293, 4308597, 4339681, 4309674, 4307824, 4302152, 4389845, 4348622, 4307994, 4326431, 4371704
**Moderate cardiovascular risk –** ***hypertension, obesity, tobacco-related diseases, alcohol-related diseases, treatment with antiplatelet or anticoagulant drugs, or uncomplicated diabetes without additional risk factors***		
Antiplatelet agents	Drug dispensing (ATC)	B01AC, C10BX02
Anticoagulants	Drug dispensing (ATC)	B01AA, B01AB, B01AE, B01AF, B01AX05
**Low cardiovascular risk –** ***absence of any of the above conditions***		

Abbreviations: ATC: Anatomical Therapeutic Chemical classification; CCAM: French classification for medical procedure; ICD-10: International Statistical Classification of Diseases and Related Health Problems 10th Revision

© 2025 Singier A et al. *JAMA Network Open*.

eTable 3. Association between exposure to angiogenesis inhibitors and arterial dissections or aneurysms in mCRC patients, by exposure period estimation method

	Cases (n = 195)	Controls (n = 1,950)	Crude OR (95% CI)	Adjusted OR[a] (95% CI)
Recommended administration schedule				
**Recency of exposure**				
Unexposed	54 (27.7)	569 (29.2)	1.00 (reference)	1.00 (reference)
Past exposure	76 (39.0)	653 (33.5)	1.29 (0.86; 1.94)	1.26 (0.84; 1.89)
Current exposure	65 (33.3)	728 (37.3)	0.95 (0.64; 1.40)	0.93 (0.63; 1.39)
**Cumulative duration of exposure**				
Unexposed	54 (27.7)	569 (29.2)	1.00 (reference)	1.00 (reference)
[2, 92] days	39 (20.0)	343 (17.6)	1.21 (0.77; 1.92)	1.17 (0.74; 1.84)
]92, 183] days	30 (15.4)	352 (18.0)	0.90 (0.56; 1.46)	0.90 (0.55; 1.47)
]183, 341] days	40 (20.5)	338 (17.3)	1.25 (0.78; 1.99)	1.26 (0.79; 2.03)
]341, 1664] days	32 (16.4)	348 (17.8)	0.96 (0.58; 1.60)	0.93 (0.56; 1.55)
Elimination time				
**Recency of exposure**				
Unexposed	54 (27.7)	569 (29.2)	1.00 (reference)	1.00 (reference)
Past exposure	45 (23.1)	549 (28.1)	0.82 (0.52; 1.29)	0.80 (0.51; 1.26)
Current exposure	96 (49.2)	832 (42.7)	1.23 (0.85; 1.78)	1.22 (0.84; 1.78)
**Cumulative duration of exposure**				
Unexposed	54 (27.7)	569 (29.2)	1.00 (reference)	1.00 (reference)
[2, 141] days	32 (16.4)	349 (17.9)	0.93 (0.56; 1.52)	0.91 (0.55; 1.49)
]141, 255] days	41 (21.0)	340 (17.4)	1.35 (0.85; 2.17)	1.33 (0.83; 2.15)
]255, 438] days	35 (17.9)	346 (17.7)	1.12 (0.68; 1.84)	1.13 (0.68; 1.87)
]438, 1997] days	33 (16.9)	346 (17.7)	1.04 (0.62; 1.75)	1.02 (0.61; 1.72)

OR: Odds Ratio, [a] adjusted for cardiovascular risk level

© 2025 Singier A et al. *JAMA Network Open*.
