# Supplementary material for: Angiogenesis Inhibitors and Arterial Dissection or Aneurysm in Patients With Metastatic Colorectal Cancer
Source: JAMA Netw Open. 2025 Dec 4;8(12):e2546960. doi: 10.1001/jamanetworkopen.2025.46960 (PMC12679322; doi:10.1001/jamanetworkopen.2025.46960)
Supplement: Supplement 2. — Data Sharing Statement [file jamanetwopen-e2546960-s002.pdf]

Data Sharing Statement

Singier. Angiogenesis Inhibitors and Arterial Dissection or Aneurysm in Patients With Metastatic Colorectal Cancer. *JAMA Netw Open*. Published December 04, 2025.
doi:10.1001/jamanetworkopen.2025.46960

Data
Data available: No

Additional Information
Explanation for why data not available: The individual-level data used in this study were obtained from the French national health insurance database and analyzed within a secure remote server environment. Access to this platform is strictly regulated and limited to authorized institutions under specific legal and ethical agreements. Due to these constraints, and in accordance with data protection regulations, it is not possible to extract or share the individual-level data outside of the secure infrastructure.
